
JOURNAL INFORMATION
==============================
NLM Title Abbreviation: ScientificWorldJournal
Journal ID: ScientificWorldJournal
NLM Journal ID: 101131163
Journal ID: TSWJ

The Scientific World Journal

ISSN: 2356-6140
EISSN: 1537-744X
Publisher: Wiley

ARTICLE INFORMATION
==============================
PMCID: PMC5917123
PMID: 16617403
DOI: 10.1100/tsw.2006.100
Article ID: 293632
Article version: 1
Subjects: Directions in Science

Syndecan-1 as a Mediator of Bacteria-Enterocyte Interactions

Henry-Stanley Michelle 1
Wells Carol L. 2 3 *
1 Department of Genetics, Cell Biology and Development, University of Minnesota, Minneapolis, 55455, USA
2 Laboratory Medicine and Pathology, University of Minnesota, Minneapolis, 55455, USA
3 Department of Surgery, University of Minnesota, Minneapolis, 55455, USA
*Carol L. Wells: wells002@tc.umn.eduAcademic Editor: Martin Goette

Publication date: 2006
Electronic publication date: 2006 Apr 14
Volume: 6
First page: 466
Last page: 471
Received 2006 Apr 3; Revised 2006 Apr 10; Accepted 2006 Apr 11
Copyright: Copyright © 2006 Michelle Henry-Stanley and Carol L. Wells.
Copyright year: 2006
License: This is an open access article distributed under the Creative Commons Attribution License, which permits unrestricted use, distribution, and reproduction in any medium, provided the original work is properly cited.
License URL: https://creativecommons.org/licenses/by/3.0/

Keywords: syndecan-1, enteric bacteria, intestinal epithelial cell

Funding: National Institutes of Health GM 066751

==============================
Directions in Science
*TheScientificWorldJOURNAL* (2006) 6, 466–471
ISSN 1537-744X; DOI 10.1100/tsw.2006.100

TheScientificWorldJOURNAL
www.thescientificworld.com

### Syndecan-1 as a Mediator of Bacteria-Enterocyte Interactions

Michelle Henry-Stanley[1] and Carol L. Wells[2,3,*]

*Departments of [1]Genetics, Cell Biology and Development, [2]Laboratory Medicine and Pathology, and [3]Surgery, University of Minnesota, Minneapolis, 55455*

**E-mails:** henry039@tc.umn.edu; wells002@tc.umn.edu

*Received April 3, 2006; Revised April 10, 2006; Accepted April 11, 2006; Published April 14, 2006*

**KEYWORDS:** syndecan-1, enteric bacteria, intestinal epithelial cell

Normal enteric bacteria frequently cause complicating infections in immunosuppressed and postsurgical patients, as well as patients with shock and trauma. Many nosocomial infections have an undefined focus and appear to be caused by translocating normal enteric bacteria that somehow penetrate the intestinal epithelium and enter otherwise sterile extraintestinal tissues[1,2,3]. Associated mortality is often high (20–40%) despite appropriate antimicrobial therapy[2,4]. Thus, clarification of the mechanisms by which normal enteric flora penetrate the intestinal epithelium is important because antibiotic therapy is often ineffective and because new knowledge may suggest novel prophylactic and therapeutic agents to decrease the costly morbidity associated with translocating enteric flora.

A variety of clinical conditions are associated with increased passage of normal enteric bacteria across the intestinal epithelial barrier. These conditions include enteric microbial overgrowth, gut atrophy, liquid diet, gut stasis, ischemia-reperfusion injury, immunosuppression, surgery, burn wounds, shock, trauma, and increased circulating endotoxin. These diverse conditions are associated with increased intestinal epithelial permeability, facilitating exposure of basolateral enterocyte surfaces, normally joined by tight junctions that prevent the paracellular passage of microbes and unwanted macromolecules (reviewed in [1]). Based on evidence that internalization of a number of bacteria is favored at the basolateral (as opposed to apical) surface of polarized epithelial cells[5,6], we recently tested the hypothesis that syndecan-1, a heparan sulfate (HS) proteoglycan (PG) prominently expressed on the basolateral epithelial surface[7,8], may be involved in interactions of normal enteric bacteria with host enterocytes[9].

The syndecans are a family of four (syndecan-1, -2, -3, -4) widely expressed transmembrane PGs. Each syndecan core protein has a transmembrane portion, a relatively short intracytoplasmic region, and an extracellular domain decorated with three to five glycosaminoglycan (GAG) chains[10,11]. Ectodomains of syndecan-1 (Fig. 1), -3, and -4 may bear both HS and chondroitin sulfate (CS) GAG chains, while syndecan-2 appears to express only HS[10,12]. Syndecan-1 is mainly/predominantly expressed on epithelia and may be found in the mesenchyme during development. Syndecan-2 is prominent in mesenchymal tissues, and on liver and neuronal cells. Syndecan-3 is associated with neural tissues and syndecan-4 is widely distributed in many cell types[10,13]. Syndecans can bind growth factors, chemokines, cytokines, extracellular matrix constituents, components of the coagulation cascade, and various microbes[14,15], and a number of elegant reviews describe aspects of syndecan structure and function[10,11,14,16].

*Corresponding author.
©2006 with author.
Published by TheScientificWorld, Ltd.; www.thescientificworld.com

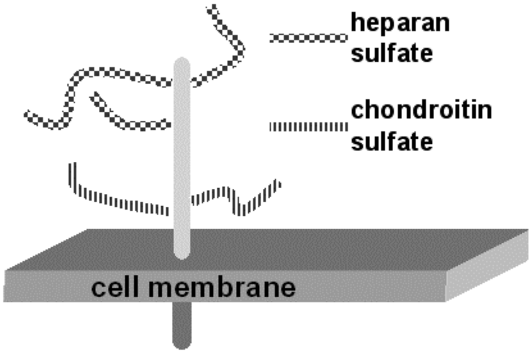

**FIGURE 1.** Diagrammatic representation of the structure of syndecan-1, a transmembrane HSPG with a comparatively short cytoplasmic domain and an ectodomain decorated with heparan sulfate and chondroitin sulfate.

Binding to host tissues is generally considered prerequisite for successful infection by a microbial pathogen. Using microbial HS-binding proteins, a variety of microbes apparently bind heparin/HS, including protozoa (*Leishmania* spp., *Plasmodium* spp., *Trypanosoma cruzi*), viruses (cytomegalovirus, herpes simplex virus, human immunodeficiency virus, pseudorabies virus), gram-negative bacteria (*Borrelia burgdorferi*, *Chlamydia trachomatis*, *Neisseria gonorrhoeae*, *Hemophilus influenza*, *Helicobacter pylori*, *Bordetella pertussis*), gram-positive bacteria (*Listeria monocytogenes*, *Staphylococcus aureus*, *Streptococcus* spp.), and acid-fast *Mycobacterium* spp.[8,15,17]. In some instances, a microbe appears to use HS as a coreceptor. For example, initial binding of herpes simplex virus to host cell HS is mediated by viral glycoprotein C, followed by fusion of virions with the host cell membrane using interactions between other viral glycoproteins and other host determinants[18,19]. Thus, HS participates in interactions of a variety of frankly pathogenic microbes with mammalian cells, but the ability of endogenous intestinal bacteria to interact with HSPGs, including syndecan-1 has received little attention.

Although there are hundreds of microbial species in the human intestine, comparatively few (e.g., *Enterococcus faecalis*, *Escherichia coli*, and other members of the family Enterobacteriaceae) are typically associated with systemic infection in high-risk patients[1,2,3,4]. To investigate the role of syndecan-1 in interactions of normal enteric flora with intestinal epithelium, we selected bacteria that might use the enterocyte as a portal of entry for systemic infection. Two enteric pathogens, *L. monocytogenes* and *Salmonella typhimurium*, known to penetrate the intestinal tract of otherwise healthy individuals, were also used as positive controls for bacterial invasion of enterocytes. Normal enteric flora included *E. faecalis, E. coli,* and other members of the family Enterobacteriaceae (*Klebsiella pneumonia*, *Citrobacter freundii*, *Morganella morganii*, *Enterobacter cloacae*, *Proteus mirabilis*). Other species included *Streptococcus bovis*, *S. agalactiae*, *S. pyogenes*, and *S. mitis*, as well as *Staphylococcus aureus* and *S. epidermidis*. *Streptococcus bovis* bacteremia and endocarditis have been associated with gastrointestinal disease, primarily colon cancer[20]. *S. agalactiae* carriage is monitored using vaginal and anorectal swabs[21]. *S. mitis* is associated with bacteremia in patients with chemotherapy-induced mucosal injury of the alimentary tract[22]. Intestinal *S. aureus*[23] and *S. epidermidis*[24] have been implicated in systemic infection in immunosuppressed patients, and *S. aureus* can translocate out of the intestinal tract of orally inoculated mice[25]. As a caveat, only one or two strains of each species were used in the study highlighted here[9], so resulting data cannot be extrapolated to define the properties of any particular species. Nonetheless, these data provide useful information about the potential scope of the interactions of syndecan-1 with normal enteric bacteria.

Of the above bacterial species, only *L. monocytogenes, S. bovis, S. agalactiae, S. pyogenes, S. mitis, S. aureus*, and *S. epidermidis* showed demonstrable binding of heparin-albumin-biotin, indicating expression of bacterial heparin-binding proteins. With exception of *S. mitis*, these species (with heparinbinding proteins) showed increased adherence to ARH-77 cells that normally have little detergent extractable HS and were transfected to express syndecan-1[26]. In this system, lack of detectable reactivity with *S. mitis* could have been due to the need for a syndecan-1 coreceptor, but this investigation was beyond the scope of the study. Fig. 2 shows an example of comparative binding of *S. pyogenes* to vector control and syndecan-1 transfected ARH-77 cells. Two polarized enterocyte cells lines were subsequently used to determine if heparin, an HS analog, could inhibit bacterial internalization by cultured enterocytes. Caco-2 enterocytes have relatively low syndecan-1 expression[8], and heparin had no noticeable effect bacterial internalization. However, using HT-29 enterocytes with prominent syndecan-1 expression[8], heparin inhibited internalization of *S. bovis*, *S. agalactiae*, *S. pyogenes*, and *S. aureus*. (Again, lack of inhibition with *S. epidermidis* and *S. mitis* could have been due to the need for a syndecan-1 coreceptor.) Results from a companion study[27] confirmed that HSPGs played an important role in internalization of *S. aureus* by HT-29 enterocytes, and this mechanism appeared different from a previously described model employing fibronectin as a mediator of *S. aureus* binding to eucaryotic cells[17,28]. Data from experiments with mutant Chinese hamster ovary cells with altered GAG expression indicated that both HS and CS (GAGs on the syndecan-1 ectodomain) participated in interactions of *L. monocytogenes*, *S. bovis*, *S. agalactiae*, *S. pyogenes*, *S. mitis*, *S. aureus*, and *S. epidermidis* with mammalian cells. These observations are summarized in Table 1.

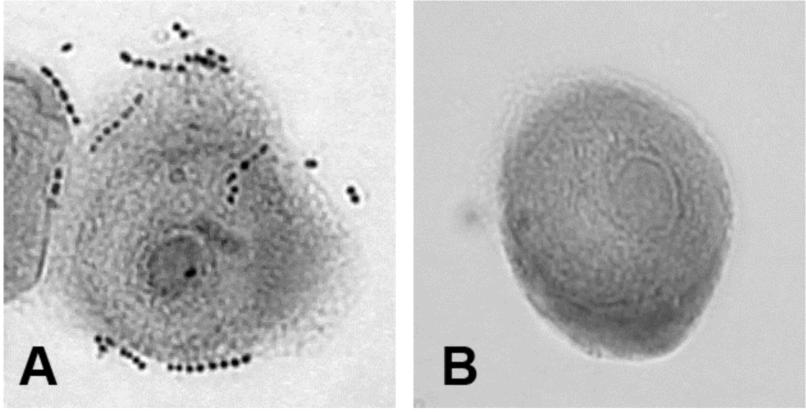

**FIGURE 2.** Phase contrast images showing adherence of many *S. pyogenes* (chains of cocci) to ARH-77 cells transfected to express syndecan-1 (A), compared to the lack of *S. pyogenes* adherence to vector control ARH-77 cell (B).

The functional assay with HT-29 enterocytes (Table 1) tested the ability of cell-bound HS to interact with enteric bacteria. Interactions with CS were not clarified, but it seems reasonable to speculate that bacterial interactions with CS-bearing syndecan-1 may be sterically hindered due to the proximity of CS to the cell membrane (Fig. 1). Interestingly, physiologically active syndecan ectodomains are constitutively shed at low levels into the extracellular milieu, shedding appears increased in wounds and in epithelium over inflamed intestinal mucosa, and shed ectodomains can compete for the same ligands as their cell surface counterparts[7,16,29]. Little is known about bacterial interactions with shed syndecan-1, and with CS itself, but there is evidence that CS interacts with *S. uberis*[30] and *B. pertussis*[31], and this is likely a fertile area for future investigation.

We originally hypothesized that intestinal syndecan-1 might provide a portal of entry for normal intestinal bacteria that cause systemic infection in high-risk patients. Although our results to date indicate that *E. faecalis*, *E. coli,* and other gram-negative enterobacteria do not interact with syndecan-1, this HSPG may mediate enterocyte interactions with some staphylococci and streptococci. Such interactions could have great importance and could affect our views regarding the use of GAG derivatives as prophylactic or therapeutic agents in specific populations of high-risk patients.

**TABLE 1**
**Summarized Results[9] of Assays Designed to Detect Bacterial Expression of Heparin-Binding Proteins and to Quantify the Ability of GAG Expression by Mutant Chinese Hamster Ovary (CHO) Cells to Modulate Bacterial Internalization, the Ability of Bacteria to Show Increased Adherence to ARH-77 Cells Transfected to Express Syndecan-1, and the Ability of Exogenous Heparin to Inhibit Bacterial Internalization by HT-29 Enterocytes (a Cell Line with Prominent Syndecan-1 Expression). *E. coli, P. vulgaris, P. mirabilis, K. pneumoniae, C. freundii, E. cloacae, S. typhimurium,* and *E. faecalis* (not included below) Gave Negative Results in All Assays.**

| Enteric Bacterial Species | Expression of Heparin-Binding Proteins | GAGs Modulate Internalization by Mutant CHO Cell Lines | Adherence to Syndecan-1-Expressing ARH-77 Cells | Heparin-Induced Inhibition of Internalization by HT-29 Cells |
|---|---|---|---|---|
| *L. monocytogenes* | + | + | + | + |
| *S. bovis* | + | + | + | + |
| *S. agalactiae* | + | + | + | + |
| *S. pyogenes* | + | + | + | + |
| *S. aureus* | + | + | + | +* |
| *S. epidermidis* | + | + | + | – |
| *S. mitis* | + | + | - | – |

*Two of three *S. aureus* strains showed heparin-induced inhibition[9,26].

### ACKNOWLEDGMENT

This work was supported in part by Public Health Service grant GM 066751 from the National Institutes of Health, USA.

### REFERENCES

1. Feltis, B.A. and Wells, C.L. (2000) Microbial translocation – Does it play a role in critical illness? *Curr. Opin. Crit. Care.* **6**, 117–122.
2. Berg, R.D. (1999) Bacterial translocation from the gastrointestinal tract. *Adv. Exp. Med. Biol.* **473**, 11–30.
3. O'Boyle, C.J., MacFie, J., Mitchell, C.J., Johnstone, D., Sagar, P.M., and Sedman, P.C. (1998) Microbiology of bacterial translocation in humans. *Gut* **42**, 29–35.
4. Pittet, D. and Wenzel, R.P. (1995) Nosocomial blood stream infections: secular trends in rates, mortality, and contribution to total hospital deaths. *Arch. Intern. Med.* **155**, 1177–1184.
5. McCormick, B.A. (2003) The use of transepithelial models to examine host-pathogen interactions *Curr. Opin. Microbiol.* **6**, 77–81.
6. Kazmierczak, B.I., Mostov, K., and Engel, J.N. (2001) Interaction of bacterial pathogens with polarized epithelium. *Ann. Rev. Microbiol.* **55**, 407–435.
7. Day, R., Ilyas, M., Daszak, P., Talbot, I., and Forbes, A. (1999) Expression of syndecan-1 in inflammatory bowel disease and a possible mechanism of heparin therapy. *Dig. Dis. Sci.* **44**, 2508–2515.
8. Henry-Stanley, M.J., Hess, D.J., Erickson, E.A., Garni, R.M., and Wells, C.L. (2003) Role of heparan sulfate in interactions of *Listeria monocytogenes* with enterocytes. *Med. Microbiol. Immunol.* **192**, 107–115.
9. Henry-Stanley, M.J., Hess, D.J., Erlandsen, S.L., and Wells, C.L. (2005) Ability of the heparan sulfate proteoglycan syndecan-1 to participate in bacterial translocation across the intestinal epithelial barrier. *Shock* **24**, 571–576.
10. Couchman, J.R. (2003) Syndecans: proteoglycan regulators of cell-surface microdomains? *Nat. Rev. Mol. Cell Biol.* **4,** 926–937.
11. Yoneda, A. and Couchman, J.R. (2003) Regulation of cytoskeletal organization by syndecan transmembrane proteoglycans. *Matrix Biol.* **22,** 25–33.
12. Deepa, S.S., Yamada, S., Zako, M., Goldberger, O., and Sugahara, K. (2004) Chondroitin sulfate chains on syndecan-1 and syndecan-4 from normal murine mammary gland epithelial cells are structurally and functionally distinct and cooperate with heparan sulfate chains to bind growth factors. A novel function to control binding of midkine, pleiotrophin, and basic fibroblast growth factor. *J. Biol. Chem.* **279**, 37368–37376.
13. Tkachenko, E., Rhodes, J.M., and Simons, M. (2005) Syndecans: new kids on the signaling block. *Circ. Res.* **96**, 488–500.
14. Park, P.W., Reizes, O., and Bernfield, M. (2000) Cell surface heparan sulfate proteoglycans: selective regulators of ligand-receptor encounters. *J. Biol. Chem.* **275,** 29923–29926.
15. Rostand, K.S. and Esko, J.D. (1997) Microbial adherence to and invasion through proteoglycans. *Infect. Immun.* **65**, 1–8.
16. Bernfield, M., Gotte, M., Park, P.W., Reizes, O., Fitzgerald, M.L., Lincecum, J., and Zako, M. (1999) Functions of cell surface heparan sulfate proteoglycans. *Ann. Rev. Biochem.* **68**, 729–777.
17. Duensing, T.D., Wing, J.S, and van Putten, J.P.M. (1999) Sulfated polysaccharide-directed recruitment of mammalian host proteins: a novel strategy in microbial pathogenesis. *Infect. Immun.* **67**, 4463–4468.
18. Feyzi, E., Trybala, E., Bergstrom, T., Lindhal, U., and Spillman, D. (1997) Structural requirement of heparan sulfate for interaction with herpes simplex virus type 1 virions and isolated glycoprotein C. *J. Biol. Chem.* **272**, 24850–24857.
19. Geraghty, R.J., Krummenacher, C., Cohen, G.H., Eisenberg, R.J., and Spear, P.G. (1998) Entry of alphaherpesviruses mediated by poliovirus receptor-related protein 1 and poliovirus receptor. *Science* **280**, 1618–1620.
20. Songy W.B., Ruoff, K.L., Facklam, R.R., Ferraro, M.J., and Falkow, S. (2002) Identification of *Streptococcus bovis* biotype I strains among *S. bovis* clinical isolates by PCR. *J. Clin. Microbiol.* **40**, 2913–2918.
21. Centers for Disease Control and Prevention (1996) Prevention of perinatal streptococcal disease: a public health perspective. *Morb. Mortal. Wkly. Rep.* **45(RR-7)**, 1–23.
22. Khan, S.A and Wingard, J.R. (2001) Infection and mucosal injury in cancer treatment. *J. Natl. Cancer. Inst. Monogr.* **29**, 31–36.
23. Squier, C., Rihs, J.D., Risa, K.J., Sagnimeni, A., Wagener, M.M., Stout, J., Muder, R.R., and Singh, N. (2002) *Staphylococcus aureus* rectal carriage and its association with infections in patients in a surgical intensive care unit and a liver transplant unit. *Infect. Cont. Hosp. Epidemiol*. **23**, 495–501.
24. Murono, K., Hirano, Y., Koyano, S., Ito, K., and Fujieda, K. (2003) Molecular comparison of bacterial isolates from blood with strains colonizing pharynx and intestine in immunocompromised patients with sepsis. *J. Med. Microbiol.* **52**, 527–530.
25. Hess, D.J., Garni, R.M., Henry-Stanley, M.J., and Wells, C.L. (2005) *Escherichia coli* modulates extraintestinal spread of *Staphylococcus aureus. Shock* **24**, 376–381.
26. Liebersbach, B.F. and Sanderson, R.D. (1994) Expression of syndecan-1 inhibits cell invasion into type I collagen. *J. Biol. Chem.* **269**, 20013–20019.
27. Hess, D.J., Henry-Stanley, M.J., Erlandsen, S.L., and Wells, C.L. (2006) Heparan sulfate proteoglycans mediate *Staphylococcus aureus* interactions with intestinal epithelium. *Med. Microbiol. Immunol.*, in press.
28. Ahmed, S., Meghji, S., Williams, R., Henderson, B., Brock, J., and Nair, S. (2001) *Staphylococcus aureus* fibronectin binding proteins are essential for internalization by osteoblasts but do not account for differences in intracellular levels of bacteria. *Infect. Immun.* **69**, 2872–2877.
29. Fitzgerald, M.L., Wang, Z., Park, P.W., Murphy, G., and Bernfield, M. (2000) Shedding of syndecan-1 and -4 ectodomains is regulated by multiple signaling pathways and mediated by a TIMP-3-sensitive metalloproteinase. *J. Cell. Biol*. **148**, 811–824.
30. Almeida, R.A., Fang, W., and Oliver, S.P. (1999) Adherence and internalization of *Streptococcus uberis* to bovine mammary epithelial cells are mediated by host cell proteoglycans. *FEMS Microbiol. Lett.* **177**, 313–317.
31. Geuijen, C.A., Willems, R.J., and Mooi, F.R. (1996) The major fimbrial subunit of *Bordetella pertussis* binds to sulfated sugars. *Infect. Immun.* **64**, 2657–2665.

**This article should be cited as follows:**

Henry-Stanley, M. and Wells, C.L. (2006) Syndecan-1 as a mediator of bacteria-enterocyte interactions. *TheScientificWorldJOURNAL* **6**, 466–471. DOI 10.1100/tsw.2006.100.

### BIOSKETCHES

**Dr. Michelle J. Henry-Stanley** is a Research Associate in the Department of Genetics, Cell Biology and Development at the University of Minnesota, Minneapolis. She obtained an M.Sc. in Laboratory Medicine and Pathology from the University of Minnesota, and a Ph.D. in Immunology and Immunopathology from the University of Arkansas for Medical Sciences, Little Rock. Her dissertation was in the laboratory of Dr. Ralph D. Sanderson and focused on the role of syndecan-1 in myeloma cell adhesion. She then completed postdoctoral studies in the laboratory of Dr. Carol Wells. Dr. Henry-Stanley's research interests center on the function of cell-surface proteoglycans and their shed counterparts in glycosaminoglycan-mediated interactions between microbes and epithelial cells.

**Dr. Carol L. Wells** is a Professor in the Department of Laboratory Medicine and Pathology, with a joint appointment in the Department of Surgery, at the University of Minnesota, Minneapolis. She received M.Sc. and Ph.D. degrees in Medical Microbiology from the University of Wisconsin, Madison. Dr. Wells did postdoctoral work with Dr. Tracey Wilkins at the Virginia Polytechnic Institute and State University in Blacksburg, Virginia, where she continued studies on the pathogenesis of selected members of the normal intestinal microflora. Dr. Wells has a long-standing research interest in the role of the normal microbial flora in the pathogenesis of complicating systemic infections in high-risk immunosuppressed, shock, and trauma patients, and she has recently expanded this work to include the role of syndecan-1 in mediating bacterial-epithelial interactions.
